# Geochemistry and Multiomics Data Differentiate Streams in Pennsylvania Based on Unconventional Oil and Gas Activity

**DOI:** 10.1128/spectrum.00770-22

**Published:** 2022-08-18

**Authors:** Maria Fernanda Campa, Jeremy R. Chen See, Lavinia V. Unverdorben, Olivia G. Wright, Kimberly A. Roth, Jonathan M. Niles, Daniel Ressler, Ella M. S. Macatugal, Andrew D. Putt, Stephen M. Techtmann, Timothy L. Righetti, Terry C. Hazen, Regina Lamendella

**Affiliations:** a University of Tennessee, Knoxville, Tennessee, USA; b Oak Ridge National Laboratory, Oak Ridge, Tennessee, USA; c Juniata College, Huntingdon, Pennsylvania, USA; d Susquehanna University, Selinsgrove, Pennsylvania, USA; e University of Guam, Mangilao, Guam, USA; f Michigan Technological University, Houghton, Michigan, USA; University of Michigan-Ann Arbor

**Keywords:** 16S rRNA, Marcellus shale, geochemistry, hydraulic fracturing, metatranscriptomics, natural gas

## Abstract

Unconventional oil and gas (UOG) extraction is increasing exponentially around the world, as new technological advances have provided cost-effective methods to extract hard-to-reach hydrocarbons. While UOG has increased the energy output of some countries, past research indicates potential impacts in nearby stream ecosystems as measured by geochemical and microbial markers. Here, we utilized a robust data set that combines 16S rRNA gene amplicon sequencing (DNA), metatranscriptomics (RNA), geochemistry, and trace element analyses to establish the impact of UOG activity in 21 sites in northern Pennsylvania. These data were also used to design predictive machine learning models to determine the UOG impact on streams. We identified multiple biomarkers of UOG activity and contributors of antimicrobial resistance within the order *Burkholderiales*. Furthermore, we identified expressed antimicrobial resistance genes, land coverage, geochemistry, and specific microbes as strong predictors of UOG status. Of the predictive models constructed (*n* = 30), 15 had accuracies higher than expected by chance and area under the curve values above 0.70. The supervised random forest models with the highest accuracy were constructed with 16S rRNA gene profiles, metatranscriptomics active microbial composition, metatranscriptomics active antimicrobial resistance genes, land coverage, and geochemistry (*n* = 23). The models identified the most important features within those data sets for classifying UOG status. These findings identified specific shifts in gene presence and expression, as well as geochemical measures, that can be used to build robust models to identify impacts of UOG development.

**IMPORTANCE** The environmental implications of unconventional oil and gas extraction are only recently starting to be systematically recorded. Our research shows the utility of microbial communities paired with geochemical markers to build strong predictive random forest models of unconventional oil and gas activity and the identification of key biomarkers. Microbial communities, their transcribed genes, and key biomarkers can be used as sentinels of environmental changes. Slight changes in microbial function and composition can be detected before chemical markers of contamination. Potential contamination, specifically from biocides, is especially concerning due to its potential to promote antibiotic resistance in the environment. Additionally, as microbial communities facilitate the bulk of nutrient cycling in the environment, small changes may have long-term repercussions. Supervised random forest models can be used to identify changes in those communities, greatly enhance our understanding of what such impacts entail, and inform environmental management decisions.

## INTRODUCTION

The economic extraction of unconventional oil and gas (UOG) has expanded the accessible global fossil fuel reserves, changing energy trade dynamics worldwide. For example, in 2019 the United States became a net exporter of energy, a feat that had not been achieved since 1952 (1). Unconventional hydrocarbon reserves are trapped in impermeable rock formations, such as shale. Horizontal drilling coupled with hydraulic fracturing (HF), a process using highly pressurized water, sand, and chemicals to fracture impermeable rocks, has facilitated the profitable extraction of UOG.

The benefits of UOG extraction are accompanied by negative impacts, including fugitive methane emissions (2), surface and groundwater ecosystem degradation (3–5), increased water scarcity (6), and wastewater handling hazards (7). The wastewater produced from the HF process, called produced and flowback water (PFW), may contain naturally occurring radioactive material from the subsurface, salt concentrations up to 7 times higher than seawater, and unknown chemical transformation products from the original proprietary blend of chemicals used to pressurize and fracture the bedrock (8). PFW can reach the environment through accidental spills or equipment failure and from incompletely treated PFW in wastewater facility effluent released into bodies of water (9).

Biocides are used in the HF process to prevent biofilm formation and corrosion due to microbial activity (10). Biocide use has been previously associated with antimicrobial resistance in other environments (11–14). Additionally, one study noted a shift in antimicrobial resistance genes in downstream sites from HF wastewater release (15). Therefore, in this study we hypothesized that UOG activity could potentially promote antimicrobial resistance genes in the surrounding ecosystems in addition to the other aforementioned environmental impacts.

Streams are particularly important environments. Headwater streams often make up 60 to 80% of the total length of a river network (16). They are also vital for nutrient cycling within their watersheds (17, 18). Headwater streams support a large diversity of wildlife to the extent that they are considered crucial to maintaining biodiversity in their larger river systems (19). Additionally, humans make use of streams, with 13.1 million people in the United States estimated to have fished in rivers and streams in 2016 (20). Stream ecosystems have previously been noted to be especially sensitive to disturbance (17). Furthermore, stream water quality can also influence well water quality through a process known as infiltration, in which stream water flows into a nearby aquifer (21). Consequently, it is important to be able to determine if UOG activity impacts nearby streams for proper environmental management, function, and protection.

Microbes are robust indicators of ecosystem quality and functioning. Past studies have evaluated their response to HF fluids, including biocides, in microcosms (22–25). Those studies showed a distinct microbial response to hydraulic fracturing fluids between samples from UOG-impacted streams and controls, as determined by diversity metrics and microbial composition and function. Microcosms have also been used been used to study the functional capabilities of microbial communities associated with produced water; for example, Borton et al. (26) utilized metabolite, metaproteomic, and metagenomic analyses, with a focus on interaction among community members and the importance of glycine betaine. Other previous studies identified geochemical markers (27) and microbial biomarkers of UOG impact (28–31) on nearby areas, while others did not find significant differences (32). The discrepancies, particularly the lack of differences found in microbial communities, may stem from the use of low-resolution operational taxonomic units (OTUs) for 16S rRNA gene data and a focus only on diversity metrics. Past studies have focused on microbial community composition and functional potential. These past studies extrapolated functional differences, leaving a knowledge gap in the identification of expressed genes. This distinction is important, as shifting microbial communities can show functional redundancy to continue supporting important environmental functions, independent of the compositional changes observed (33).

While past studies have expanded our understanding of UOG impacts on geochemistry and microbial community structures of streams, none of them has coupled metatranscriptomics with geochemical markers to understand the functional differences related to UOG proximity. Metatranscriptomics analysis reveals the active genes and the microbes expressing them. We sought to determine if UOG activity impacted the microbes present and their gene expression. Functional gene information can help expand our understanding of long-term environmental impacts of UOG extraction. We also investigated potential differences in various geochemistry and water quality measurements based on proximity to UOG activity.

Here, we hypothesized that there is a relationship between UOG activity and microbial community structure and gene expression (including antimicrobial resistance genes [ARGs] due to the stress caused by proximity to UOG practices and the chemicals used in HF, such as biocides), and geochemical markers in headwater streams and streambed sediments.

Furthermore, these high-resolution data sets can be utilized to build predictive random forest (RF) models that infer UOG impacts, which can be applied to other UOG locations to identify universal microbial biomarkers of UOG activity. To test these hypotheses, we combined 16S rRNA gene profiling, metatranscriptomics, geochemistry, and trace element analyses to establish the impact of UOG activity in 21 sites in northern Pennsylvania streams.

## RESULTS AND DISCUSSION

### Site description, geochemical properties, and ecosystem function indicators.

**FIG 1:**
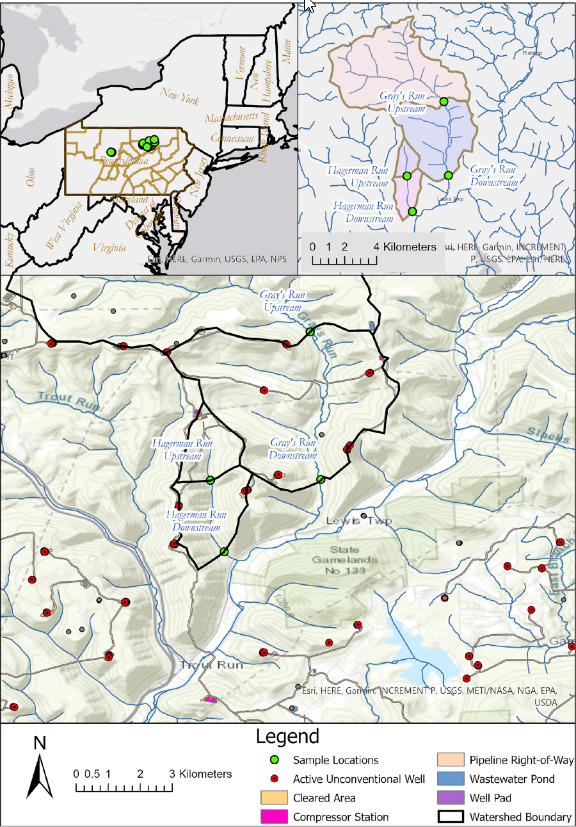
Map of all streams sampled in Pennsylvania for this study. The map shows sampling location, well pads location, cleared area, location of compressor stations, the pipeline right-of-way, wastewater pond, and watershed boundaries.

Samples were taken from 21 sites in northern Pennsylvania (Fig. 1) during the summer of 2019. Of these, 9 were classified as UOG^−^ (no well pads with active wells present in the watershed) and 12 were classified as UOG^+^ (at least one well pad with at least one active well present in the watershed). Only active wells present in the watershed were included in the well count number for UOG^+^ streams. However, Hagerman Run Upstream was still considered UOG^+^ due to being downstream of a compressor station and adjacent to a haul road used for HF activities, despite having no active wells in its watershed, and Alex Branch Run and Little Laurel Run were classified as UOG^+^ principally because they both had been previously impacted by HF fluid spills (34). We utilized a watershed-level approach, collecting samples upstream and downstream of UOG activity, to determine if UOG activity impacted the ionic and trace metal markers in a stream.

Two data sets were used for all analyses (16S sediment, 16S water, ARG sediment, gene sediment, and metatranscriptome sediment): (i) a data set with all samples, named UOG, which included samples from all 21 sites, and (ii) a subset, named PAIRED, which contained data from streams where upstream and downstream sampling from UOG activity was conducted to help minimize differences stemming from comparing multiple watersheds (see Table S1 in the supplemental material). Furthermore, to test if balancing the data set yielded better models, another data set with an equal number of UOG^+^ and UOG^−^ samples, named BALANCED, was created and used for RF models and total AMR contributors only.

**FIG 2:**
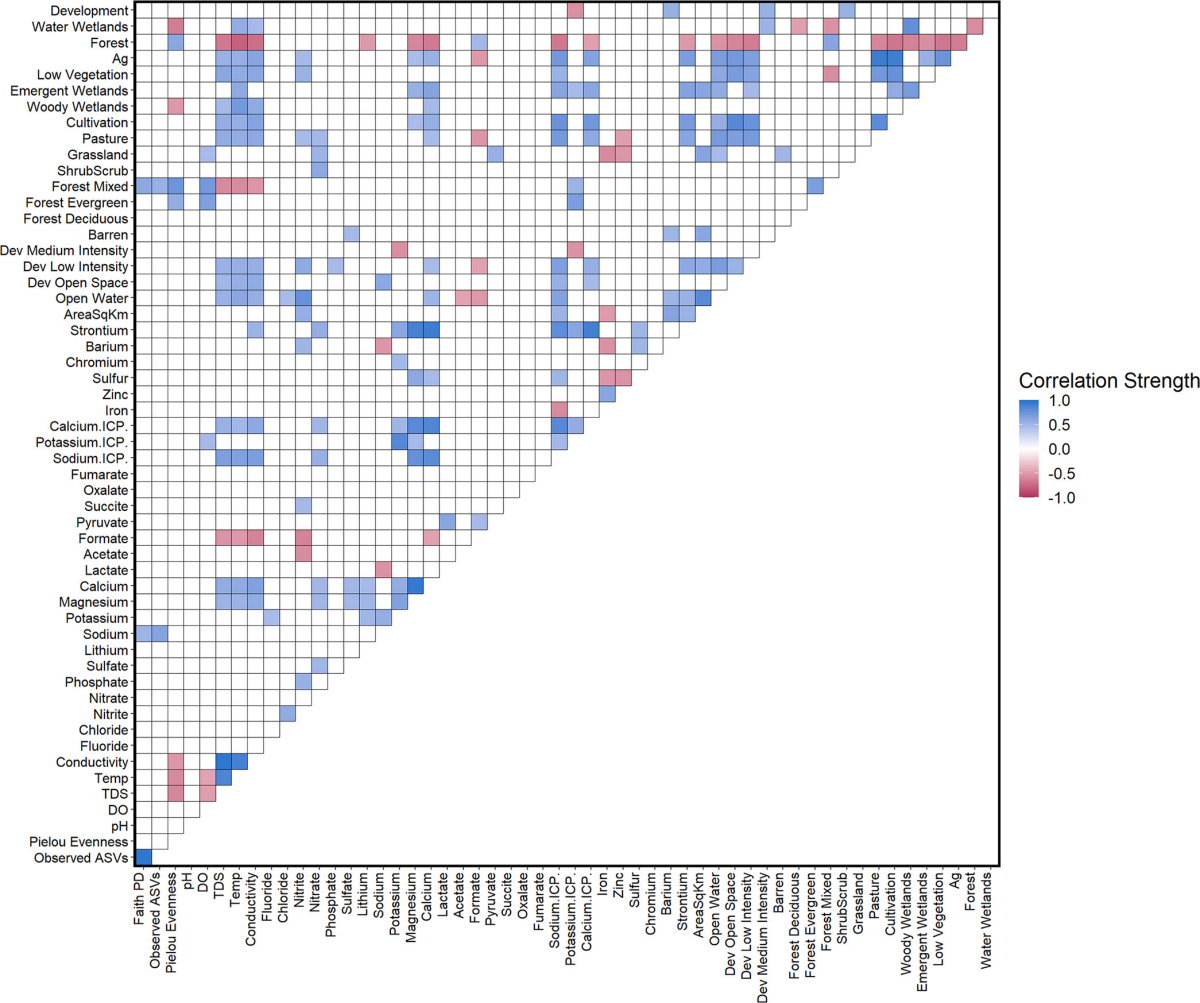
Correlogram of sample metadata showing significant Spearman rank correlations. Correlations were calculated using the water UOG data set. The square colors show whether the correlation was positive (blue) or negative (red), with darker squares indicating stronger correlations.

Significant differences (Wilcoxon rank sum test, *P* ≤ 0.05) were identified for the mean of each geochemistry and water quality measure within each data set based on UOG status. Stream water temperature (UOG^+^ = 16.0°C; UOG^−^ = 13.8°C), total dissolved solids (TDS) (UOG^+^ = 50.16 mg/liter, UOG^−^ = 26.63 mg/liter), and conductivity (UOG^+^ = 77.867 μS, UOG^−^= 43.100 μS) were higher in UOG^+^ streams than UOG^−^ streams. Within the water UOG and PAIRED data sets, a total of eight terrain and land cover variables were significantly higher (Wilcoxon rank sum test, *P* ≤ 0.05) in UOG^+^ samples: area (UOG^+^ = 37.117 m^2^, UOG^−^ = 2.552 m^2^, PAIRED UOG^+^ = 41.812 m^2^, PAIRED UOG^−^ = 1.098 m^2^), Water_11 (UOG^+^ = 0.161 m^2^, UOG^−^ = 0 m^2^, PAIRED UOG^+^ = 0.128 m^2^, PAIRED UOG^−^ = 0), Barren_31 (UOG^+^ = 0.231 m^2^, UOG^−^ = 0.063 m^2^, PAIRED UOG^+^ = 0.328 m^2^, PAIRED UOG^−^ = 0), Dev_LI_22 (UOG^+^ = 0.042 m^2^, UOG^−^ = 0), Grassland (UOG^+^ = 0.412 m^2^, UOG^−^ = 0.248 m^2^, PAIRED UOG^+^ = 0.428 m^2^, PAIRED UOG^−^ = 0), Pasture_81 (UOG^+^ = 8.160 m^2^, UOG^−^ = 2.249 m^2^), For_Everg_ (PAIRED UOG^+^ = 3.655 m^2^, PAIRED UOG^−^ = 0.120 m^2^), and For_Mix_43 (PAIRED UOG^+^ = 18.460 m^2^, PAIRED UOG^−^ = 8.487 m^2^). Six of these eight terrain and land cover variables corresponded to the presence of materials and spaces made by humans (see Table S2), indicating a different landscape between UOG statuses. Significant correlations were also present among elements and ions and other metadata based on the water UOG data set (Fig. 2).

A comparison between UOG^+^ and UOG^−^ data within the sediment UOG data set (all reported concentrations are in milligrams per liter, except Sulfate is in μM) showed that sulfate (UOG^+^ = 6.961, UOG^−^ = 5.063), Cl (UOG^+^ = 3.879, UOG^−^ = 1.754), Na (UOG^+^ = 14.300, UOG^−^ = 9.838), Mg (UOG^+^ = 2.585, UOG^−^ = 1.325), Ca (UOG^+^ = 23.368, UOG^−^ = 8.452), Na determined by inductively coupled spectroscopy (Na ICP; UOG^+^ = 5.560, UOG^−^ = 2.527), and S (UOG^+^ = 2.588, UOG^−^ = 1.874) were significantly higher in UOG^+^ sediments, while Zn (UOG^+^ = 0.015, UOG^−^ = 0.432) and F (UOG^+^ = 0.076, UOG^−^ = 0.094) were higher in UOG^−^ sediments (Wilcoxon rank sum test, *P* ≤ 0.05). In the PAIRED data set, sediment samples showed that Mg (PAIRED UOG^+^ = 2.709, PAIRED UOG^−^ = 1.116), sulfate (PAIRED UOG^+^ = 7.051, PAIRED UOG^−^ = 5.667), Ca (PAIRED UOG^+^ = 26.141, PAIRED UOG^−^ = 8.230), and Sr (PAIRED UOG^+^ = 0.127, PAIRED UOG^−^ = 0.060) were significantly higher in UOG^+^ downstream sediment (see Table S2). Additionally, the comparison between UOG^+^ and UOG^−^ in the water UOG data set revealed that the concentrations of Ba (UOG^+^ = 0.061, UOG^−^ = 0.008), S (UOG^+^ = 1.291, UOG^−^ = 0.810), Na ICP (UOG^+^ = 2.185, UOG^−^ = 0.465), Sr (UOG^+^ = 0.032, UOG^−^ = 0.012), and sulfate (UOG^+^ = 4.530, UOG^−^ = 3.173) were significantly higher in UOG^+^ water, with Fe (UOG^+^ = 0.014, UOG^−^ = 0.226) and Zn (UOG^+^ = 0, UOG^−^ = 0.001) significantly higher in UOG^−^ water. The PAIRED stream samples showed that Sr (PAIRED UOG^+^ = 0.038, PAIRED UOG^−^ = 0.009), K (PAIRED UOG^+^ = 0.762, PAIRED UOG^−^ = 0.282), S (PAIRED UOG^+^ = 1.406, PAIRED UOG^−^ = 0.705), Ca ICP (PAIRED UOG^+^ = 9.076, PAIRED UOG^−^ = 2.529), and Na ICP (PAIRED UOG^+^ = 2.390, PAIRED UOG^−^ = 0.530) were significantly higher in UOG^+^ water.

Others have found that Na, Ca, Cl, Li, B, Br, Sr, and Ba are effective tracers of produced water (35–38). Higher conductivity has also been associated with UOG activity, as shales contain high levels of brines (4). Marcellus shale has characteristic Na-Ca-Cl brines due to old and evaporated seawater (4). Therefore, as observed in our sediment samples, elevated concentrations of Na-Ca-Cl in UOG^+^ sediment, and particularly between upstream and downstream comparisons of the same stream (Ca ICP and Na ICP in water PAIRED samples) are strong geochemical indicators of a significant impact from Marcellus shale extraction activity in the area.

Our Ca, Cl, and Na measurements were similar to those reported in other studies of the impact of UOG activity on nearby streams. Our Na ICP and Ca ICP values were similar to those from Mumford et al. (32), in which samples were also collected from headwater streams overlying a Marcellus shale formation, with different proximities to UOG activity (Na = 2.26 mg/liter, Ca = 8.13 mg/liter). Several (*n* = 9: 6 UOG^+^, 3 UOG^−^) of our Cl values were higher than the maximum value reported by Mumford et al. of 100.09 mg/liter (32). Additionally, we found significant differences among Na ICP and Ca ICP water measurements based on UOG status, while Mumford and colleagues did not (32). This and other differences in geochemistry results could possibly be due to samples being grouped simply based on the presence of UOG activity (active well pads) upstream in our study, instead of by degree of impact, as in the Mumford et al. study. Interestingly, the Cl values in this study were roughly comparable to those reported in a study that collected samples downstream of a fracking fluid injection site (Akob et al. [39]) (Cl = 218.53 mg/liter). However, the Akob et al. study reported much higher Na and Ca values than ours (Na = 60.94 mg/liter, Ca = 31.31 mg/liter) (39), indicating that these UOG^+^ streams were likely less impacted by UOG than the stream analyzed by Akob et al. Similarly, higher Na, Ca, and Cl values were observed in a study in North Dakota that took multiple measurements over time in a stream following a HF spill (Cozzarelli et al. [38]) (Na = 371 mg/liter, Ca = 115.5 mg/liter, Cl = 117.60 mg/liter). Therefore, the elevated NA ICP and Ca ICP measurements in the UOG^+^ samples in our study suggest that those streams were impacted.

### Microbial and functional composition and relationship to chemical and land use parameters.

16S rRNA gene sequencing revealed a total of 43,044 amplicon sequence variants (ASVs), providing a higher-resolution view (compared to OTUs) of microbial diversity in these stream ecosystems. The Faith’s phylogenetic diversity metric differed significantly between water UOG^+^ and water UOG^−^ samples in the water UOG data set (see Table S3). The number of unique ASVs was significantly higher (*P* ≤ 0.05, Kruskal-Wallis) in UOG^−^ water and sediment samples in the UOG data sets and sediment PAIRED data sets compared to their respective UOG^+^ samples in those data sets. In contrast, the active microbial composition data showed no significant differences in observed features and Pielou’s evenness between UOG status in either the UOG or PAIRED data sets. Lower alpha diversity has been previously associated with UOG activity (28, 30, 39) though not consistently, as other studies have found no significant differences in alpha diversity (29, 31, 32).

**FIG 3:**
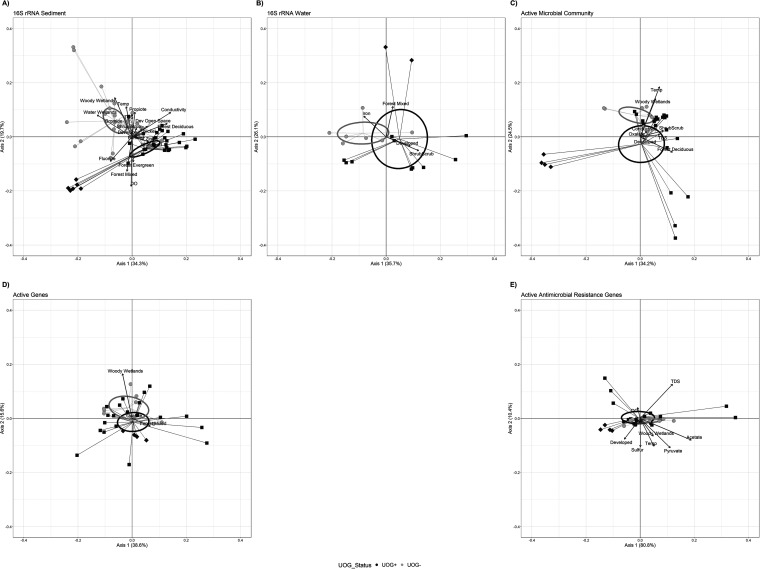
Principal coordinates analyses (PCoA) with vectors, indicating significant environmental parameters and 95% confidence intervals around the centroids, with lines connecting the centroids to their respective samples. Dark gray, UOG^+^ samples; light gray, UOG^−^ samples. Circles, UOG^−^ samples; squares, UOG^+^ samples, with the exception of samples from the two spill sites (ABR and LLR), which are shown with diamonds. UOG data sets are visualized in all panels. (A) Weighted Unifrac distance of total bacterial community, 16S rRNA gene-derived ASVs from streambed sediment. (B) Weighted Unifrac distance of 16S rRNA gene-derived ASVs from stream water. (C) Bray-Curtis dissimilarity of active microbial community, metatranscriptomics of streambed sediment. (D) Bray-Curtis dissimilarity of active genes, metatranscriptomics of streambed sediment. (E) Bray-Curtis dissimilarity of active antimicrobial resistance genes, metatranscriptomics of streambed sediment.

Beta diversity analysis of the bacterial community composition revealed differential clustering of UOG^+^ and UOG^−^ sediment samples (Fig. 3A) (weighted UniFrac, permutational analysis of variance [PERMANOVA], *P* ≤ 0.05). Conversely, the clustering of water samples based on impact status was not significant (Fig. 3B) (weighted UniFrac, PERMANOVA, *P* > 0.05). Interestingly, water and sediment samples from the UOG^+^ spill sites (Alex Branch Run and Little Laurel Run) formed a unique cluster away from other samples. The metatranscriptomics active microbial community analyses revealed significant differences between UOG groups for both the UOG (Fig. 3C) (Bray-Curtis dissimilarity, PERMANOVA, *P* = 0.05) and PAIRED upstream and downstream comparison data sets (PERMANOVA, *P* ≤ 0.05), with Alex Branch Run and Little Laurel Run samples clustering away from the other samples. However, based on PERMANOVA, it was determined that the gene dissimilarity (Fig. 3D) (Bray-Curtis dissimilarity, PERMANOVA, *P* > 0.05) and ARG-only dissimilarity (Fig. 3E) (Bray-Curtis dissimilarity, PERMANOVA, *P* > 0.05) metatranscriptomics profiles were not significantly different between UOG^+^ and UOG^−^. Beta diversity was visualized for these five data sets using principal coordinates analysis (PCoA) plots (Fig. 3). In all data sets, geochemical, land use, and organic acid parameters in the data sets significantly interacted with UOG status and contributed to bacterial variation (see Table S4). 16S rRNA sediment comparisons had the greatest number of explanatory parameters, with 19 in total, indicating that bacterial community composition was more affected by these factors than the active (metatranscriptomics) community and expressed functional genes (Fig. 3). Br and Ba levels significantly explained microbial community variation among the total bacterial sediment community in conjunction with UOG impact status. These results agree with results of other studies that found Br and Ba were present at elevated concentrations downstream of oil and gas wastewater release (7, 36, 38). These results also showed that Br and Ba accumulation in sediment can alter microbial communities, which could have an effect on ecosystem function, such as nutrient cycling. Additionally, conductivity, TDS, temperature, dissolved oxygen (DO), propionate, Ni, fluoride, and Zn also significantly explained variation (PERMANOVA, *P* ≤ 0.05) when coupled with UOG impact status and could potentially serve as markers for UOG-impacted streams. Among UOG^+^ samples, the number of wells significantly explained variation in the genes sediment data set, and well pads significantly explained variation for that data set in addition to the metatranscriptome data set (see Table S5).

While landscape had an effect on differences in microbial community function and composition, the results presented here indicate it is not the only factor. However, a limitation of the current study is the age of the land coverage database, as it was last updated in 2011 (40), while sampling was conducted in 2019. Another limitation is that well density alone might not fully explain the effects caused by other UOG development parameters, such as pipeline infrastructure and spills (41).

### Active community composition differs more than functional gene expression.

Both microbial composition and function differed based on UOG status; however, variation was more pronounced in active composition profiles, as seen in the PCoA plots (Fig. 3) and the significant PERMANOVA results compared to the gene expression profiles. Environmental microbial communities are complex adaptive systems, and system dynamics might allow for microbial community composition to shift while bulk functional profiles stay the same due to functional redundancy, functional resilience, stability of function, and/or resistance to disturbance (42). Thus, as microbial composition is disturbed, different community members with versatile physiologies may adapt to perform functions needed for system survival (33, 42). In this system, bacterial community composition (16S rRNA genes) can be used to determine UOG activity near streams, supporting previous findings (28, 30, 31). In contrast, Bray-Curtis dissimilarity metrics of both active genes and ARG data sets showed no significant (PERMANOVA, *P* > 0.05) clustering between UOG statuses (see Table S3). These differences and the variable results of others (28–32) suggest that diversity alone is not a good marker of UOG activity and should be combined with other metrics and modeling.

### Biomarkers of UOG activity.

Features (microbes and genes) that differed significantly in abundance based on UOG status, i.e., biomarkers of UOG activity, were identified using linear discriminant analysis (LDA) of effect size (LEfSe) and ANOVA-like differential expression (ALDEx2). Multiple overlapping UOG^−^ biomarkers were identified in the 16S sediment UOG (see Fig. S1A) and 16S sediment PAIRED samples (see Fig. S1B). Active sediment UOG (see Fig. S1C) and active sediment PAIRED data sets (see Fig. S1D) shared several biomarkers with a few exceptions. In the water data sets, 16S water UOG (see Fig. S2A) identified two overlapping UOG^+^ biomarkers, while 16S water PAIRED (see Fig. S2B) identified multiple overlapping UOG^−^ biomarkers and only one UOG^+^. Taxonomic UOG status predictors from the total and active microbial sediment community indicated that while there was some overlap, namely, *Alphaproteobacteria*, such as *Xanthobacteraceae*, *Reyranellaceae*, and *Rhodobiaceae* being strong indicators of UOG^−^ (see Fig. S1A to D), different biomarkers emerged as indicators of UOG^+^. Furthermore, a greater percentage of the active microbial sediment community was identified as differential compared to the total sediment community (UOG active community, 0.272%; PAIRED active community, 0.472%; UOG total community, 0.198%; PAIRED total community, 0.198%). However, both total and active microbial communities could be used to identify multiple *Betaproteobacteria* biomarkers of UOG^+^, specifically, members of *Burkholderiales.* The order *Burkholderiales* is ubiquitous in stream ecosystems (43) and has been found in high concentrations in other UOG^+^ streams in Pennsylvania (30) and in other streams impacted by anthropogenic activity, as *Burkholderiales* seem to degrade and thrive in the presence of complex organic compounds (44, 45); this may explain why members such as *Rubrivivax*, *Ramlibacter*, *Methylbium*, *Pelistega*, *Polaromonas*, and *Inhella* are all active biomarkers of UOG activity (see Fig. S1C and D). The consistency of these bacterial assemblages, compounded with their increased activity in UOG streams, suggest members of *Burkholderiales* could be useful indicators for determining UOG impact.

Both ARG and active genes had very few UOG biomarkers identified compared to total and active microbial community composition data sets. Active gene biomarkers were not detected in either the UOG or PAIRED data sets based on ALDEx2. In contrast, 8 biomarkers were identified in the UOG data set (see Fig. S3) and 5 were identified in the PAIRED data set (see Fig. S4) using LEfSe. Large subunit ribosomal proteins (L28 and L35) were identified in UOG and PAIRED data sets, indicating that protein synthesis was highly expressed in UOG^+^ samples. Large subunit ribosomal proteins have a role in peptide bond formation and have an active role in the bacterial translation stress response to nutrient depletion, toxins, and antimicrobials (46). Bacteria in UOG wastewater have previously shown an active stress response due to nutrient depletion, high salt content, extreme pH, and biocide usage (47, 48). Another biomarker of UOG^+^ status was the pilus assembly protein CpaE. Pili are common among bacteria, but their expression in UOG^+^ is of interest, as pili are involved in adhesion, biofilm formation, motility, DNA uptake, and pathogenicity (49). Biofilm formation is common in UOG wastewater and is a recurrent issue that can cause fouling and equipment failure due to microbiologically induced corrosion (50). Biofilms in UOG equipment and wastewater have also been shown to have a higher tolerance to biocides commonly used in UOG practices (50), and wastewater from Marcellus shale UOG activity has been shown to carry transcripts for biofilm formation (47).

An additional enzyme, tyrosinase, an oxidoreductase related to pigmentation (51), was also upregulated in UOG^+^. The increased expression of tyrosinase and large subunit ribosomal protein L35 may be related to the high expression of *Burkholderiales*, as their functional profile overexpressed those two enzymes. Moreover, the PAIRED data set comparison also identified bacterioferritin, another oxidoreductase, as a biomarker of UOG^+^. Bacterioferritin can sequester Fe and protect the cell from oxidative stress (52). Iron acquisition has been previously identified as a genetic marker present in UOG wastewater (48). The upregulation of both tyrosinase and bacterioferritin may indicate the UOG^+^ cells were under more oxidative stress, which could have been caused by increased metal concentrations. This can be corroborated by levels of metals such as Mg, Zn, and Sr, which were detected at higher levels in UOG^+^ sites (*P* ≤ 0.05).

Similarly, ARG biomarkers were not identified with ALDEx2; however, when we used LEfSe, 4 ARG biomarkers were identified in the UOG data set (see Fig. S5) and 5 biomarkers were identified in the PAIRED data set (see Fig. S6). The rifampin-resistant beta subunit of RNA polymerase was identified in both UOG and PAIRED data sets as a biomarker of UOG^+^, whereas beta-lactam class B beta-lactamases were identified as biomarkers of UOG^−^ in both data sets. Rifampin resistance has been found previously in the environment, as *Actinomycetes* naturally produce rifampin in the environment; thus, this resistance gene may also be carried by multidrug-resistant microbes (53). In contrast, macrolide-resistant 23S rRNA mutation and aminoglycoside-resistant 16S were biomarkers of both UOG^+^ and UOG^−^, indicating that those genes are ubiquitous in the environment or are contributed by a common anthropogenic source.

### Identification of microbial contributors to the ARG profile of streams.

Differential genes, taxa, and stream characteristics were also identified by examining feature importance from the RF models. The most predictive ARG types were macrolide-resistant 23S rRNA mutation, aminoglycoside-resistant 16S ribosomal subunit protein, rifampin-resistant beta subunit RNA polymerase, tetracycline-resistant 16S ribosomal subunit protein, multidrug-resistant 23S rRNA mutation, phenicol-resistant 23S mutation (MEG5781), and pactamycin-resistant 16S ribosomal subunit protein. The top contributors vary by ARG, but the core contributors of ARG were the same in both UOG statuses; these included *Acidobacteria*, *Actinobacteria*, *Alphaproteobacteria*, *Burkholderiales*, *Firmicutes*, and *Gammaproteobacteria*, among others (see Table S6).

Analysis of the number of taxa expressing ARGs in the BALANCED data set revealed that 6,431 different microbes had at least one ARG in UOG^+^, while 6,317 had at least one in UOG^−^. In contrast, 4,726 taxa expressed ARGs in at least 70% of the UOG^+^ samples, while 4,641 taxa contributed to ARG expression in 70% of the UOG^−^ samples. However, the difference in the number of taxa expressing ARGs was not significant (Wilcoxon rank sum test, *P* > 0.05).

### Functional profile of *Burkholderiales*, a group identified as UOG biomarkers and carrier of ARGs.

**FIG 4:**
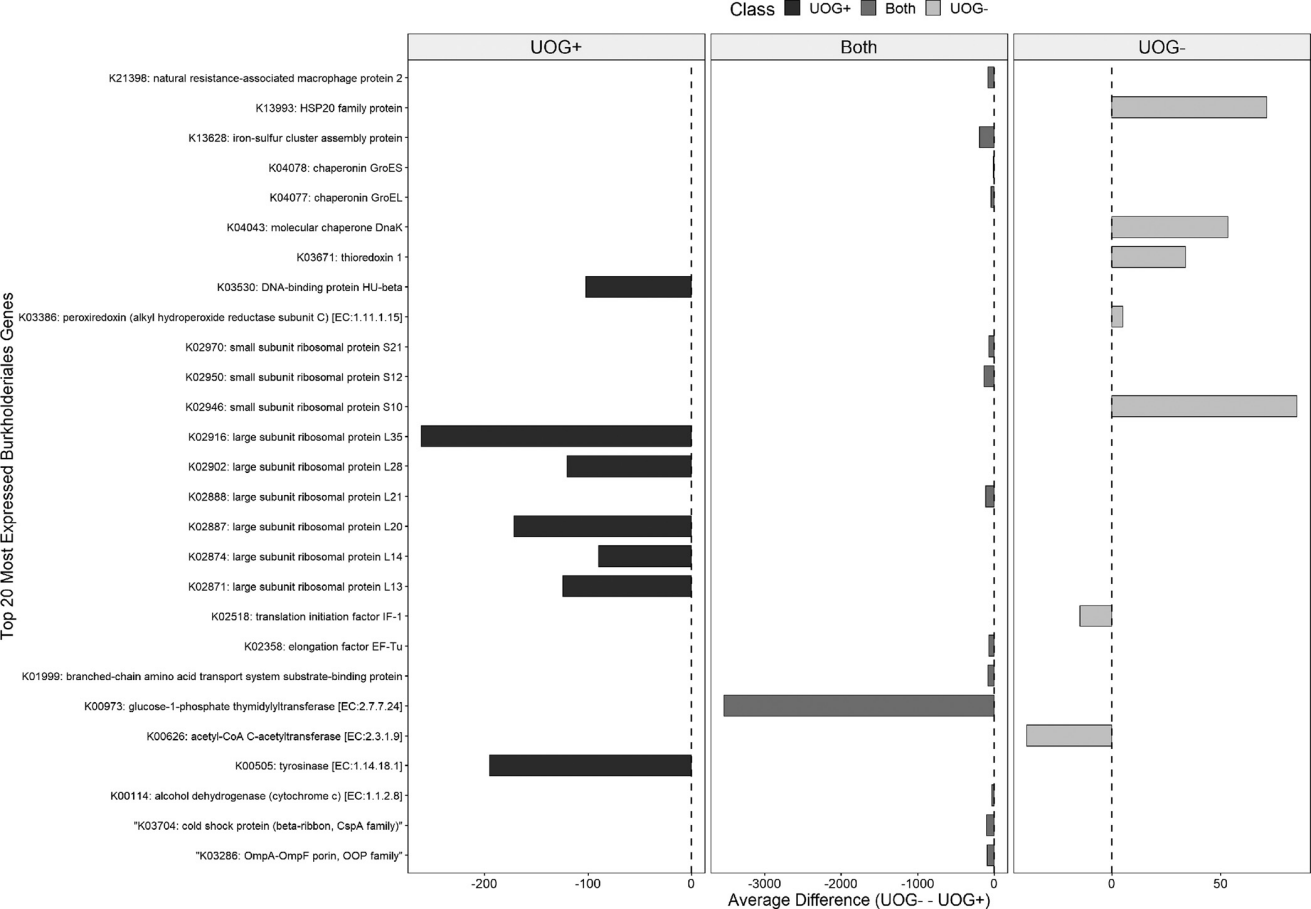
*Burkholderiales* top 20 most expressed genes in UOG^+^ (left) and UOG^−^ (right), with genes highly expressed in both shown in the middle. The difference between each gene’s average normalized (based on counts per minute) expression in UOG^−^ from its average in UOG^+^ is shown on the *x* axis. Therefore, negative values indicate higher expression in UOG^+^, while positive values indicate higher expression in UOG^−^. Axes are not consistent across panels. Several of the differences in expression were significant (see Table S7 in the supplemental material).

Members of the family *Burkholderiales* were identified as biomarkers for UOG^+^ activity based on ALDEx2 and LEfSe results. A functional profile of its members was built to identify expressed genes and determine if expression differed based on UOG status. The top 20 most highly expressed genes in *Burkholderiales* UOG^−^ and UOG^+^ are shown in Fig. 4. Thirteen of the top 20 active genes overlapped in both UOG statuses. Multiple large subunit ribosomal proteins were identified among the most expressed in UOG^+^ *Burkholderiales*. Specifically, L35 (K02916), L28 (K02902), L27 (K02899), L20 (K02887), and L13 (K02871) coding sequences were among the most expressed in UOG^+^ but not UOG^−^. Among the 20 most active genes in both statuses, large subunit ribosomal protein L35 (K02916), iron-sulfur cluster assembly protein (K13628), large subunit ribosomal protein L20 (K02887), large subunit ribosomal protein L13 (K02871), and small subunit ribosomal protein S12 (K02950) were significantly more highly expressed in UOG^+^ (see Table S7).

The UOG^+^ *Burkholderiales* ARG profile included 51 different ARGs, and the UOG^−^ *Burkholderiales* ARG profile had 41 different ARGs. The difference between the number of *Burkholderiales* expressed ARGs in UOG^+^ and UOG^−^ samples was not significant (Wilcoxon rank sum test, *P* > 0.05). Additionally, the overall UOG^−^ *Burkholderiales* top ARG profile was very similar to the UOG^+^ ARG profile, with the same 10 ARGs being the most highly expressed. Still, we identified more ARGs in the UOG^+^ *Burkholderiales* functional profile than in the UOG^−^ profile, indicating that environmental and/or anthropogenic stressors may contribute to the ARG profile of *Burkholderiales*. While *Burkholderiales* members harbor intrinsic ARGs, *Burkholderiales* was of particular interest as it contains many common soil bacteria and many opportunistic human pathogens, making the detected levels of ARGs a concern (54). Higher trace metal concentrations have been associated with higher ARG expression in the environment, and in these streams Mg and Sr were detected at higher levels in UOG^+^ streams.

### Random forest models can successfully identify UOG-impacted samples.

**FIG 5:**
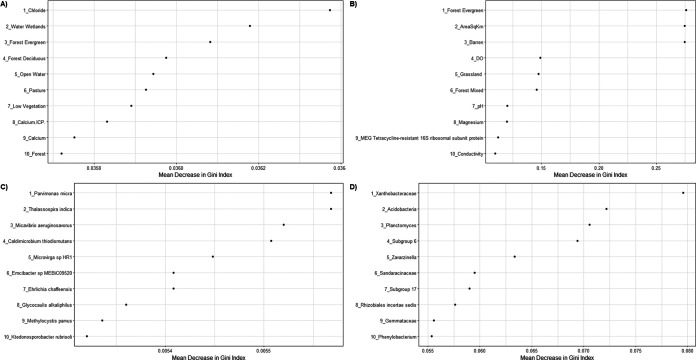
The four random forest models with the highest overall predictive accuracy for their input data. The top predictors of unconventional oil and gas status for each model are listed; the *x* axis represents the mean decrease in Gini index for each predictor, and the *y* axis lists the top 10 predictors for each model. (A) The BALANCED land cover and geochemistry model with metadata, overall accuracy 100%. (B) The PAIRED active antimicrobial resistance genes and metadata model, overall accuracy of 96.27%. (C) The BALANCED active microbial composition model, overall accuracy 93.45%. (D) The 16S rRNA gene amplicon ASVs UOG sediment model, overall accuracy 89.87%. These four models had AUC values >0.89.

Thirty RF models were constructed, in which features (ASVs, active species, expressed genes, or expressed ARGs, depending on the data set) were used as separate variables to try to correctly classify samples’ UOG statuses. Models were evaluated using accuracy, i.e., how many times they correctly classified samples. They were also evaluated using area under the curve (AUC) values for precision-recall (PR) and receiver operating characteristic (ROC) curves to directly take into account misclassifications. An AUC value of at least 0.70 for ROC curves is generally considered indicative of a good model (55). We also used 0.70 as a cutoff for relevant PR curve values. Models were constructed with geochemistry and land coverage data alone, total bacterial community, active microbial community, functional genes expression profile, and ARGs (see Table S8). Overall accuracy was expected to be around 70% or better based on RF modeling done in a previous study (30), and most models did yield >70% overall accuracy. However, only 17 of the models had accuracy higher than expected by chance for both classes, and of those, 15 had AUC values above 0.70 for both curves. The failure of many models was likely due to imbalanced sample sizes, i.e., having more UOG^+^ then UOG^−^ samples in most data sets. This was supported by the overall improved performance of the water UOG model after samples from another study (56) were added to increase the number of UOG^−^ samples, with accuracy for the worse-performing class increased from 11.7% to 34.3% (see Table S8). Additionally, models made with microbial composition data derived from metatranscriptomics data performed better than those made with active genes (see Table S8). Still, models made with expressed ARGs and metadata performed very well too, having accuracies and AUC values all above 0.90 (see Table S8). Furthermore, to test if balancing the data set yielded better models, the BALANCED data set, with an equal number of UOG^+^ and UOG^−^ samples, was also used to create RF models. Considering accuracy alone, the four best models were the BALANCED land coverage and geochemistry, PAIRED ARGs sediment with metadata, BALANCED active microbial composition, and UOG 16S rRNA gene sediment models, with BALANCED models only including upstream (UOG^−^) and their matched downstream samples. The best predictors for the models are shown in Fig. 5, and all predictors for all models are shown in Table S9.

Microbial composition RF models are very versatile and have been used in the past to predict 26 geochemistry parameters, like uranium in groundwater (57), and in up to 20 distinct geographical locations across the ocean (58). RF models have also been able to predict past contamination (e.g., an oil spill) even though geochemical signatures have returned to baseline measurements, indicating a lasting effect in microbial communities (57). In this study, ARG, land cover and geochemistry data, active microbial composition, and bacterial community composition were the best inputs to create predictive RF models for UOG activity. RF models have been used previously to predict UOG statuses of sediment samples in a longitudinal 5-year study (30), and those samples were used to create the RF model yielding a 68.6% overall accuracy (30). Conversely, another study that only used sediment data had a higher performance, 82.03% (31), but in that instance the model was constructed using only taxa identified by LEfSe as differentially abundant. This indicated the accuracies of our sediment models, using either land coverage and geochemistry alone (UOG 82.92%, PAIRED 86.33%, BALANCED 100%), ASVs alone, (89.87% overall accuracy), a combination of land coverage, geochemistry, and ASVs (UOG 89.34%, PAIRED 89.26%, BALANCED 82.53% accuracy), active microbial composition (PAIRED 91.07% and BALANCED 93.45%) or with land coverage and geochemistry (UOG 92.16% and PAIRED 89.23%), and ARGs and land coverage and geochemistry (UOG 95.91%, PAIRED 96.27%, BALANCED 96.13% accuracy) had the highest performance in UOG activity prediction (see Table S8). Surprisingly, genes detected through metatranscriptomics were not effective inputs for the model. This could have been caused by an overwhelming prevalence of housekeeping genes, or genes needed for system function, independent of the environmental pressures. Overall, this study showed that RF can harness differences in environments to predict UOG status at a finer scale than standard diversity metrics.

This study showed that UOG activity has an impact on land coverage, geochemistry, total and active microbial community, and ARGs that together can be used to predict UOG status of streambed sediment. These models can be used as a monitoring tool to assess UOG activity impacts even when geochemical signatures have not drastically changed. In addition, biomarker taxa and expressed genes identified in this study could help facilitate the future development of quantitative PCR or chromatin immunoprecipitation-based assays for the rapid detection and prioritization of watersheds impacted by UOG activity.

## MATERIALS AND METHODS

Details of our methods pertaining to sampling design, site selection, and land cover are provided in Text S1 in the supplemental material.

### Water chemistry.

Water quality (TDS, DO, conductivity, pH, and temperature) was also measured at each site using a YSI Pro Plus probe. Anions, cations, and organic acids in water and sediment pore water were measured using a Dionex ICS 5000+ instrument (Thermo Fisher Scientific, Waltham, MA, USA) as described in the supplemental material. A list of all ions can be found in Table S10. Dissolved trace elements were measured at the University of Tennessee Water Chemistry Core Facility using an inductively coupled plasma optical emission spectrometer (ICP-OES) iCAP 7400 system (Thermo Fisher Scientific) following EPA method 200.7 rev 4.4 (59). As an internal check, Ca, K, and Na were measured using both IC and ICPOES; samples measured with ICP are labeled X_ICP. The Wilcoxon rank sum test was used to determine statistical significance (α = 0.05) based on UOG status.

### Nucleic acid extraction, 16S rRNA gene amplification, and sequencing.

DNA was extracted using a ZymoBiomics DNA Micro Prep kit, and RNA was extracted using a ZymoBiomics RNA Mini Prep kit according to the manufacturer’s specifications (Zymo Research, Irvine, CA, USA). At each site, triplicate sediment samples were collected for a total of 63 samples. All three samples from each site were used for 16S analysis, while only two were used for metatranscriptomics. For sediment samples, 0.25 g of sediment was used as input. A single water sample was collected from each site by pushing 200 to 600 mL of water through a 0.2-μm membrane filter (Millipore Sigma, Burlington, MA, USA). The entire membrane was then used as input for extraction. The V4 region of the 16S rRNA gene was amplified using primers and conditions previously described (60), and the libraries were prepared as previously described (23). See Text S1 for primers, thermocycler conditions, library preparation, and sequencing conditions. Data generated from sequencing were downloaded and imported into QIIME2-2019.7 (61) for preprocessing, diversity, and biomarker analysis (see Text S1).

### Metatranscriptomics library preparation, sequencing, and data processing.

Metatranscriptomic libraries were prepared with extracted RNA using the NEBNext Ultra II RNA library kit for Illumina (New England Biolabs, Ipswich, MA, USA). See Text S1 for additional details on library preparation, purification, quality checking, and sequencing. Raw data were processed and then annotated for community composition with Kraken2 (62) and for community function with Emapper (63). Diversity and biomarker analyses are detailed in Text S1.

### Random forest analysis.

RF analysis was performed using R with a normalized feature table, along with water quality and geochemistry for each data set. The R package caret (64) was used to partition 66% of the data for training the RF model and the remainder for testing. The random forest package (65) was then used with the training data to create a model to predict sample impact status and calculate the predictive importance of each feature, as measured by Gini decrease. The model’s effectiveness was evaluated with the test data. Overall accuracy as well as Gini decreases were recorded. AUC values were calculated for both precision-recall (PR) and receiving operating characteristic (ROC) curves with the PRROC package (66). This process was repeated 1,000 times to obtain average accuracy and average Gini decreases and AUC values for each of the data sets, both with and without metadata (16S sediment UOG, 16S sediment PAIRED 16S water UOG, 16S water PAIRED, ARG sediment UOG, ARG sediment PAIRED, genes sediment UOG, genes sediment UOG, microbial composition sediment UOG, and microbial composition sediment PAIRED). Notably, two upstream sites (Hagerman’s Run and Lower Gray’s Run) were classified as UOG^+^. Therefore, for each of the sediment data sets, the BALANCED data sets consisted of only samples that had matched UOG^−^ upstream and UOG^+^ downstream were also used for random forest modeling, i.e., samples from Hagerman’s Run and Lower Gray’s Run were excluded from the BALANCED data sets due to those streams’ upstream sites being classified as UOG^+^.

### Data availability.

16S rRNA gene and metatranscriptomics sequence data are available from the NCBI Short Read Archive database under BioProject PRJNA664393. Random forest code is available at https://github.com/jcbioinformatics/MultiomicsFrackingSupplemental/tree/main/RandomForestCode, and the random forest input files are available from the authors upon request.
