# Supplementary material for: Geochemistry and Multiomics Data Differentiate Streams in Pennsylvania Based on Unconventional Oil and Gas Activity
Source: Microbiol Spectr. 2022 Aug 18;10(5):e00770-22. doi: 10.1128/spectrum.00770-22 (PMC9603415; doi:10.1128/spectrum.00770-22)
Supplement: Supplemental file 1 — Supplemental material. Download spectrum.00770-22-s0001.pdf, PDF file, 1.1 MB [file spectrum.00770-22-s0001.pdf]

Title: Geochemistry and multiomics data differentiate streams in Pennsylvania based on unconventional oil and gas activity

Authors: Maria Fernanda Campa*[a,b], Jeremy R. Chen See*[c], Lavinia V. Unverdorben[c], Olivia G. Wright[c], Kimberly A. Roth[c], Jonathan M. Niles[d], Daniel Ressler[d], Ella M. S. Macatugal[e], Andrew D. Putt[a,b], Stephen M. Techtmann[f], Timothy L. Righetti[e], Terry C. Hazen[#a,b], Regina Lamendella[#c]

[a] University of Tennessee, Knoxville, TN
[b] Oak Ridge National Laboratory, Oak Ridge, TN
[c] Juniata College, Huntingdon, PA
[d] Susquehanna University, Selinsgrove, PA
[e] University of Guam, Mangilao, GU
[f] Michigan Technological University, Houghton, MI

*co-first authors
[#]co-corresponding authors

Supplemental Methods

Site Selection and Sampling Design. Only active wells present in the watershed were included in the well count number for UOG+ streams. However, Hagerman Run Upstream was denoted as UOG+ due to being downstream of a compressor station and adjacent to a haul road used for fracking activities, despite having no active wells in its watershed, and Alex Branch Run and Little Laurel Run were classified as UOG+, as they both had been previously impacted by fracking fluid spills (1).

Site selection of streams was performed on streams using ArcGis 10.7 (ESRI). The watershed pour method was used to determine watershed boundaries and sizes for each sample site. Oil and gas data from the Pennsylvania Department of Environmental Protection was gathered from the Pennsylvania Spatial Data Access (PASDA) database and was used to obtain UOG gas well layer and information,

which was subsequently mapped with the watersheds. Well presence was confirmed with Google Earth, with well pads then digitized from the satellite imagery and overlaid onto the watershed layer as polygons. If any portion of a well pad was in the watershed, it was considered part of the watershed due to the possibility of spills and contaminants across the well pad. Final sampling sites were determined based upon the presence of UOG gas wells and ability for access, with several streams allowing for matched sampling upstream and downstream of UOG activity (either public lands or permission obtained from private landowners) and to create a gradient of UOG+ in different watersheds. In addition, a detailed land use analysis was performed for each watershed in ArcGis 10.7 using the National Land Cover Database (2) to determine if there were differences among land use in UOG- and UOG+ sample sites.

PASDA data for oil and gas extraction site locations were imported into ArcGIS Pro. A stream model for unassessed waters in PA was imported as well. ArcGIS's watershed tool was then used with that model to create a polygon of the watershed encompassing each sampling location. Watersheds were manually inspected. The size of each watershed was then determined (AreaSqKm). Site coordinates and UOG Status were added the watersheds' characteristics. Land cover data for Pennsylvania from the 2011 National Land Cover Database (NLCD) was then imported. Land cover information for each site's watershed was then calculated based on the land cover within its polygon. Several summary fields were calculated as well based on the NLCD data (see Supplemental Methods Table 1). Wells and well pads were both selected based on overlap with the watersheds' polygons and included in the count for that site. Lastly, distance from sample site to the nearest well pad was calculated using ArcGIS's measuring tool. A description of the land characteristics measured can be found at https://www.mrlc.gov/data/legends/national-land-cover-database-2011-nlcd2011-legend

Ions, Organic Acids, Metals, and Trace Elements Measurements. Water samples were filtered in the field through a 0.2 µm Millipore Express® polyethersulfone (PES) membrane filter (Millipore Sigma, Burlington, Massachusetts, USA), while the sediment samples were processed to collect pore-water to serve as a proxy of sediment ions, organic acids concentration, metals, and trace elements (3). Pore-water was collected by placing 20 g in a filter device (Amicon Ultra 100 K MWCO, Millipore Sigma) and centrifuging at 3,000 g at 4°C for 1 hour. One mL of sample was aliquoted on vials for ions and organic

acid analyses. Cation samples were acidified by adding 1 M HCl so that HCl made up 10% of the final volume. Samples were stored in a -20°C freezer until analyses. Dionex Seven Anion Standard II (Thermo Fisher Scientific, Waltham, Massachusetts, USA), Dionex Six Cation-II Standard (Thermo Fisher Scientific), and in-house organic acid standards were used to establish calibration curves (0.1- 50 mg/L for Anions and Cations, and 0.5-200 µM for organic acids). For metals and trace elements measurements, 5 mL of filtered stream water or sediment pore water were acidified with 1.5% 12M HCl. Standards were purchased (SPEX CertiPrep, Metuchen, New Jersey, USA) and calibration curves were established from 0.1- 50 mg/L. The ions measured were Ammonium, Bromide (Br), Calcium (Ca), Chloride (Cl), Fluoride (F), Lithium (Li), Magnesium (Mg), Nitrate, Nitrite, Phosphate, Sodium (Na), Sulfate, and Potassium (K). The organic acids measured were Acetate, Butyrate, Formate, Fumarate, Lactate, Oxalate, Propionate, Pyruvate, and Succinate. The metal and trace elements measured were Arsenic (As), Barium (Ba), Calcium (Ca), Chromium (Cr), Cobalt (Co), Iron (Fe), Lead (Pb), Nickel (Ni), Potassium (K), Sodium (Na), Strontium (Sr), Sulfur (S), and Zinc (Zn). Spearman rank correlations among all metadata were calculated through R (4) with the hmisc package (5) and visualized with the ggcorrplot package (6) using data for the water UOG samples.

16S rRNA Amplicon Library Preparation. Briefly, 25 µl reactions including 12.5 µl of 10 µM Phusion Flash High-Fidelity PCR Master Mix (Thermo Fisher Scientific), 0.5 µl of 10 µM 515F primer (GTGCCAGCMGCCGCGGTAA), 0.5 µl of 10 µM 806r barcoded primer (GGACTACHVGGGTWTCTAAT) that incorporated Illumina adapters and barcoded sequences, and ~50 ng of template DNA (7). The thermocycler parameters were set at a 3 min 94°C ramp followed by 35 cycles of 45 sec at 94.0°C, 60 sec at 50.0°C, and 90 sec at 72.0°C (7). Finally, temperature was held for 10 min at 72.0°C and maintained at 4.0°C until the samples were removed from the thermocycler.

PCR products were visually inspected with a gel, making sure negative controls did not show signs of amplification. Non-target amplicons were removed using Zymo Select-a-Size kit following manufacturer specifications (Zymo Research, Irvine, California, USA). Amplicons were then checked for quality using a DNA 1000 kit (Agilent Technologies, Santa Clara, California, USA) with an Agilent 2100 Bioanalyzer (Agilent Technologies). The amplicons were quantified using a Qubit 3.0 fluorometer and

combined in equimolar concentrations. The final concentration was determined using quantitative PCR (qPCR) using the NEBNext Library Quant Kit for Illumina (New England Biolabs, Ipswich, Massachusetts, USA).

The final 4 nM library was sequenced using V2 chemistry to produce 2 x 150 bp sequences (300 cycle) on an Illumina MiSeq at Oak Ridge National Laboratory.

16S rRNA Gene Data Processing. Both forward and reverse reads were filtered with a length of 150 bp and an expected error of 0.5. Sequences were grouped into amplicon sequence variants (ASVs) using the DADA2 pipeline (8). Taxonomy was assigned to ASVs using a Naïve Bayes classifier, with the Silva 132 SSURef NR99 515F/806R region sequences database (9), in QIIME2 (10). A rooted phylogenetic tree was generated with MAFFT (11) and FastTree2 (12) as implemented in QIIME2's "qiime phylogeny align-to-tree-mafft-fasttree" command. ASVs identified as chloroplast or mitochondria were removed on the basis that they likely represented eukaryotic organelle contamination, instead of bacteria. Samples that had fewer than 1500 sequences after this filtering were removed, resulting in the exclusion of two sediment and two water samples. Samples were split into two datasets per analysis type: UOG and PAIRED, with the UOG datasets containing all samples collected for that analysis and the PAIRED datasets containing only samples from streams with both upstream and downstream sites. A cumulative sum scaling (CSS)-normalized table (13) was created for each dataset for use with random forest and beta diversity analyses. Normalization steps for all datasets are summarized in Supplemental Methods Table 2.

Additional data were obtained from https://www.ncbi.nlm.nih.gov/bioproject/PRJNA707295 (14) to increase the number of UOG- water samples for random forest modelling. Raw sequencing data for their fourth sampling event (summer 2019) were processed identically to the samples collected in this study and then merged with the water UOG dataset ASV table for use with random forest modeling. The merged ASV table was filtered to retain only ASVs that were present in both studies.

Diversity Calculations and Biomarker Analysis. Alpha diversity was calculated and analyzed using the unnormalized table through QIIME2. This table was then rarefied, with 10 steps, 20 iterations per step, and a step size equal to the minimum depth. See Supplemental Methods Table 3 for minimum and maximum rarefaction depths for all datasets. The Faith's Phylogenetic Diversity (15), Observed Features, and Pielou's Evenness (16) metrics were used for each dataset. Significance was assessed using a Kruskal-Wallis pairwise test with impact status as the class. Alpha diversity values were visualized using ggplot2 (17) in R. Spearman correlations were calculated between alpha diversity metrics and wells for both UOG datasets for all three metrics using rstatix (18) in R.

The Weighted Unifrac distance metric (19) was used to calculate dissimilarity among samples based on the CSS-normalized ASV table. A Principal Coordinates Analysis (PCoA) plot was then generated using the resulting distance matrix through the phyloseq package (20) in R, with 95% standard error confidence intervals being created with the ggordiplots package (21). A PERMANOVA test was also conducted using the distance matrix to evaluate the significance of clustering based on fracking status (UOG+ vs. UOG-). PERMANOVA (Adonis) was also used to identify metadata (geochemistry, water quality, and land cover measurements) that significantly explained variation among the communities in conjunction with impact status. These steps were performed for each dataset within QIIME2. All p-values are listed in Supplemental Table 4. Adonis was also used to see how much variation could be explained by the number of wells and well pads among the UOG+ samples for both water and sediment UOG datasets.

Biomarker analysis was conducted using LEfSe (LDA Effect Size) (22) and ALDEx2 (ANOVA-Like Differential Expression) (23). LEfSe was used to identify differentially abundant taxa (Kruskal-Wallis, $\alpha=0.05$) and quantify their enrichment (linear discriminant analysis) based on CPM-normalized abundances of a species-level collapsed ASV table. Consequently, LEfSe evaluated taxa at the species through Domain levels. ALDEx2 was used through QIIME2 with collapsed ASV tables, with tables being collapsed from the Phylum through Species levels. Both LEfSe and ALDEx2 were used with all data sets.

Metatranscriptomics Library Preparation. Metatranscriptomics libraries were quality checked using an Agilent 2100 BioAnalyzer and the Agilent DNA High Sensitivity DNA kit (Agilent Technologies). The

libraries were then pooled with equimolar concentration and gel purified on a 2% agarose gel using the Qiagen QIAquick Gel Purification kit (Qiagen) to size select between 250-400 bp. The purified library was sequenced using a 2 x 150 bp Illumina kit on a HiSeq 4000 at the DNA Technologies Core at University of California-Davis.

Metatranscriptomics Data Preprocessing. The quality of the raw data was evaluated with FastQC (24). The data were then filtered using fastp (25) with a sliding window of 4 and a minimum average Phred Q score of 28. Sequences shorter than 90 bp were discarded. A k-mer based approach, Kraken2 (26), was subsequently used to annotate the filtered sequences with a version of its standard database that included fungi genomes from RefSeq (27) in addition to the standard libraries (RefSeq Archaea, RefSeq Bacteria, RefSeq plasmid, RefSeq virus, GRCh38 human assembly (28), and UniVec core (29)). Species level annotations were compiled in a table for downstream analyses, except for *Homo sapiens* to avoid human contamination impacting results. Sequences classified as *Homo sapiens* were removed from the filtered sequence files, which were then paired using PEAR (30), with a minimum overlap of 10 and a p-value cutoff of 0.05. The paired files were then dereplicated with VSEARCH (31) at 100% identity, and the sequence abundance annotations were added to the headers using the "--sizeout" flag. Emapper v2.0 (32) was run on the dereplicated sequences with version 5 of the eggNOG database (33). Hits against KEGG Orthologs were then used, along with the original abundances of the query sequence. Those abundances were RPK-normalized based on the lengths of the KEGG Orthologs in the Emapper database by dividing the abundances by the length of the database sequence it was annotated as and then multiplying the quotient by 1000.

Dereplicated paired sequences were also used with BLAST (34) and the MEGARes 2.0 (35) database to examine antimicrobial resistance. The maximum number of target sequences was set to 1, with a maximum e-value of 0.001. Only hits that had an alignment length of at least 60 bp and a percent identity of at least 90% were considered. A table was created with the RPK-normalized counts for all remaining hits. For all three datasets (metatranscriptome, expressed genes, and antimicrobial resistance), tables containing only samples with paired upstream and downstream samples were created and used for analysis as well.

Metatranscriptomics Data Analyses. Alpha diversity analyses conducted through QIIME2 as with the 16S rRNA gene amplicon datasets, except only the Observed Features and Pielou's Evenness metrics were used. Beta diversity analyses were also once more done through QIIME2, with the only differences being the Bray-Curtis distance metric (36) was used instead and the tables were previously re-normalized with the counts per million (CPM) method, instead of CSS. Likewise, random forest analyses were conducted as before, except the tables were CPM-normalized beforehand. LEfSe was run as before using the RPK-normalized tables as input. Values in the RPK-normalized tables were first rounded to the nearest integer in preparation for Aldex2 due to that program not tolerating decimals. Aldex2 was then performed as with the 16S datasets. Adonis was performed again as before with all metatranscriptomic datasets. Spearman correlations were calculated between alpha diversity metrics and wells for each UOG dataset for both metrics using rstatix in R.

Functional Profile of *Burkholderiales*. KO annotations belonging to sequences identified as taxa within *Burkholderiales* via Kraken2 were extracted from the CPM-RPK-normalized table. The resulting table was then split into two, one for UOG+ samples and one for UOG- samples. Within each table, the sum of each gene's abundance for all samples was calculated and used to determine which ones were the most expressed by *Burkholderiales*. Those most expressed genes were visualized and tested for significance (Wilcoxon Rank Sum test, $p \leq 0.05$) through R. This process was repeated for the ARG dataset to determine *Burkholderiales'* most expressed ARGs. The number of ARGs expressed by *Burkholderiales* was additionally examined but found not to significantly differ based on UOG Status (Wilcoxon Rank Sum test).

Identification of Microbial Contributors to the ARG Profile. The corresponding sequence header information for each ARG (specifically the NCBI taxonomy id added by Kraken2) was used to identify the bacteria responsible for its expression. This information was added to the CPM-RPK-normalized table, stratifying it, with rows in the form of ARG;NCBI taxonomy id. The table was split into two: one for UOG+ samples and the other for UOG-. ARGs were then extracted to individual tables so that each ARG had

both a UOG+ and UOG- table. NCBI taxonomy ids were converted to lineages using TaxonKit (37) and the normalized abundance information was retained from the initial stratified table and the abundances were then converted to relative abundance (https://github.com/jcbioinformatics/MultiomicsFrackingSupplemental/tree/main/S.Table6).

Watershed Comparisons. PERMANOVA tests were used with the UOG distance matrix for each dataset to see if samples clustered significantly based on watershed (HUC8, HUC10, and HUC12). Watershed classifications were obtained from PASDA (38). Due to only having a single sample representative of the watershed, water samples from Alex Branch Run, Brunnerdale Run, Elk Creek Upstream, Hoagland Branch Middle, and Little Laurel Run were omitted from HUC12 analysis, and Alex Branch Run and Little Laurel Run were also omitted from HUC8 and HUC10 analysis. PERMANOVA tests were used with the distance metrics for the five UOG datasets to determine if samples clustered significantly by any of these watershed classifications.

Supplemental Methods Table 1. Land cover summary field formulas.

Field	Calculation
LowVegetation	21_Dev_OS + 71_Grassland
Agriculture	81_Pasture + 82_Cultivat
Forest	41_For_Decid + 42_For_Everg + 43_For_Mix
WaterWetland	11_Water + 90_Woody_Wet + 95_Emerg_Wet
Developed	22_Dev_LI + 23_Dev_MI + 31_Barren + 52_ShrubScrub

Supplemental Methods Table 2: Normalization steps prior to each type of analysis for all datasets. Expressed Genes (KOs) and Expressed ARG datasets were normalized using the same methods.

Data Type	Analysis	Normalization
16S rRNA	Alpha Diversity	Rarefaction
16S rRNA	Beta Diversity	Cumulative Sum Scaling
16S rRNA	LEfSe	Counts per Million

16S rRNA	Random Forest	Counts per Million
16S rRNA	ALDEx2	Centered Log-Ratio (internal)
MT	Active Composition Alpha Diversity	Rarefaction
MT	Active Composition Beta Diversity	Counts per Million
MT	Active Composition LEfSe	Counts per Million
MT	Active Composition Random Forest	Counts per Million
MT	Active Composition ALDEx2	Centered Log-Ratio (internal)
MT	*Burkholderiales* Functional Profile	Counts per Million
MT	Expressed Genes Alpha Diversity	Reads per Kilobase (RPK) + Rarefaction
MT	Expressed Genes Beta Diversity	RPK + Counts per Million
MT	Expressed Genes LEfSe	RPK + Counts per Million
MT	Expressed Genes Random Forest	RPK + Counts per Million
MT	Expressed Genes ALDEx2	RPK + Centered Log-Ratio (internal)

Supplemental Methods Table 3: Minimum and maximum rarefaction depths per sample

Dataset	Minimum Depth	Maximum Depth	Step_Size
16S UOG Sediment	700	7000	700
16S PAIRED Sediment	700	7000	700
16S UOG Water	1040	10400	1040
16S PAIRED Water	1040	10400	1040
ARG UOG Sediment	27950	279500	27950
ARG PAIRED Sediment	27950	279500	27950
KEGG UOG Genes Sediment	15140	151400	15140
KEGG PAIRED Genes Sediment	15140	151400	15140
Kraken2 UOG Sediment	251070	2510700	251070
Kraken2 PAIRED Sediment	251070	2510700	251070

Results

Watershed Comparisons. Significant differences among watersheds were detected (https://github.com/jcbioinformatics/MultiomicsFrackingSupplemental/blob/main/S.Table1.xlsx). Therefore, the inclusion of the analysis based on the PAIRED datasets would have lessened the influence of watershed-level variation as we investigated differences due to hydraulic fracturing status.

Geochemical Properties and Ecosystem Function Indicators. Within the UOG dataset, size of watershed (AreaSqKm), open water (11_Water), barren (31_Barren), developed low intensity (22_Dev_LI), grassland, and pasture/hay (81_Pasture/Hay) were significantly higher in UOG+ samples (Wilcoxon rank sum test, $p<0.05$). The PAIRED dataset (which included only samples from streams with upstream and downstream sites) also had six land cover measurements that differed between UOG status (Wilcoxon rank sum test, $p <0.05$). As observed with the full dataset, watershed size, barren, grassland, and open water area were higher in UOG+ sites, in addition to barren land (31_Barren), but evergreen forest (42_For_Everg), and forest mixed (43_For_Mix) were now significantly higher in UOG+ sites as well. See https://github.com/jcbioinformatics/MultiomicsFrackingSupplemental/blob/main/S.Table2.xlsx

Wells and Well Pads. None of the alpha diversity metrics in the five datasets (using only UOG+) were significantly correlated with the number of wells or well pads (Spearman, $p>0.05$). However, the number of wells significantly explained variation among the UOG+ samples in the expressed genes dataset, and the number of well pads significantly explained variation for both that dataset and the metatranscriptome (active composition) dataset (https://github.com/jcbioinformatics/MultiomicsFrackingSupplemental/blob/main/S.Table5.xlsx).

Supplemental Figures

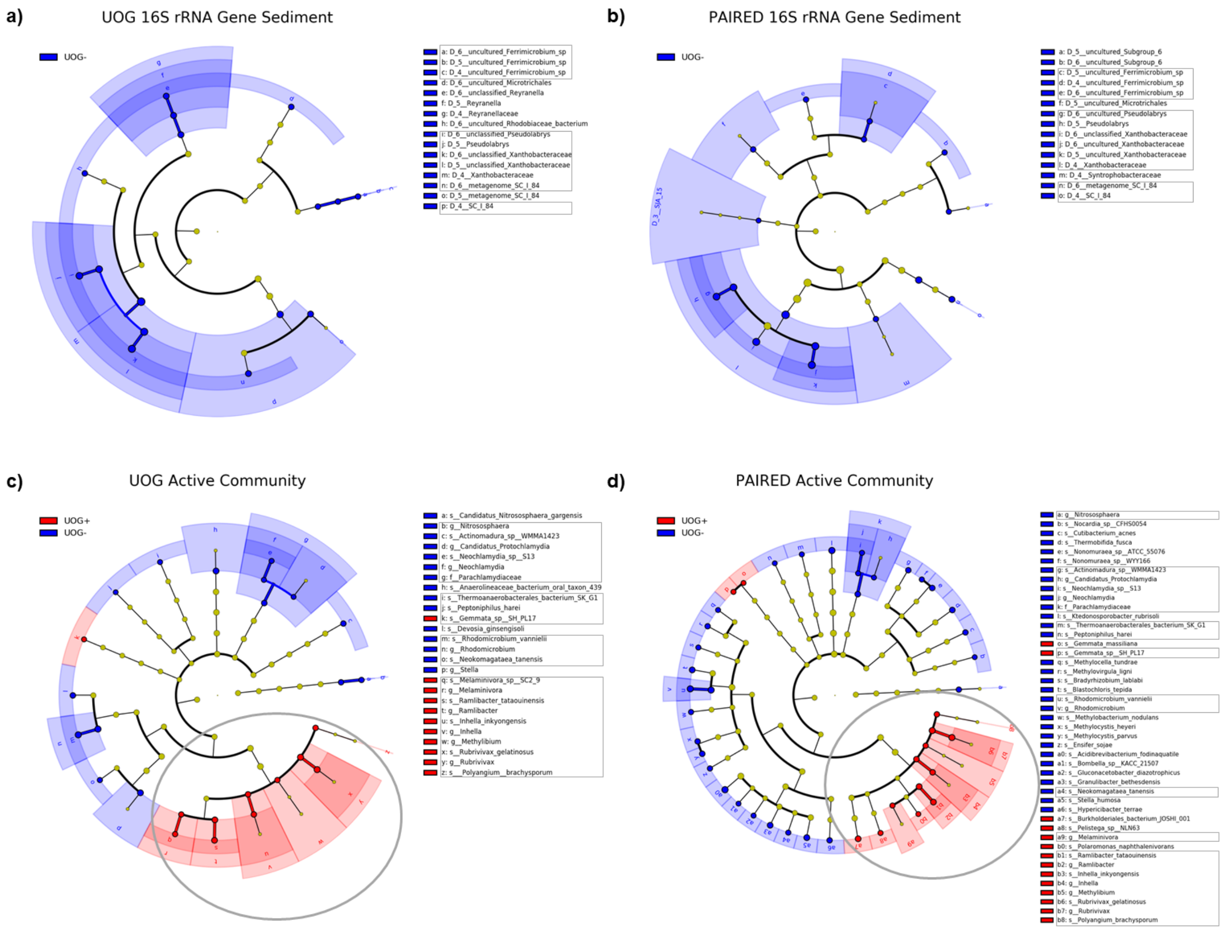

Supplemental Figure 1. Biomarkers of unconventional oil and gas (UOG) activity. A) 16S rRNA gene sediment dataset, only biomarkers UOG- were consistently identified by ALDEx2 and LEfSe, i.e. both programs identified them as being differential, B) 16S rRNA gene PAIRED sediment dataset, only biomarkers UOG- were consistently identified by ALDEx2 and LEfSe, C) metatranscriptomics active microbial community dataset, ALDEx2 and LEfSe both identified biomarkers of UOG- and UOG+ sites, D) metatranscriptomics active microbial community PAIRED dataset, ALDEx2 and LEfSe both identified biomarkers of UOG- and UOG+ sites. Blue represents UOG- biomarkers, and red represents UOG+ biomarkers. Yellow nodes represent parent taxa that were included in the cladogram but were not identified as enriched themselves. Shading was used in cases where multiple differential features overlapped, i.e. both a taxon and at least one of its children taxa were identified as enriched. The leaves

that are bolded contain differential taxa. The grey circles encompass the taxa within *Burkholderiales* that were identified as being differentially active in the metatranscriptome datasets, and the grey boxes encompass taxa that were enriched in both UOG and PAIRED datasets for 16S rRNA gene (a and b) and metatranscriptome analysis (c and d).

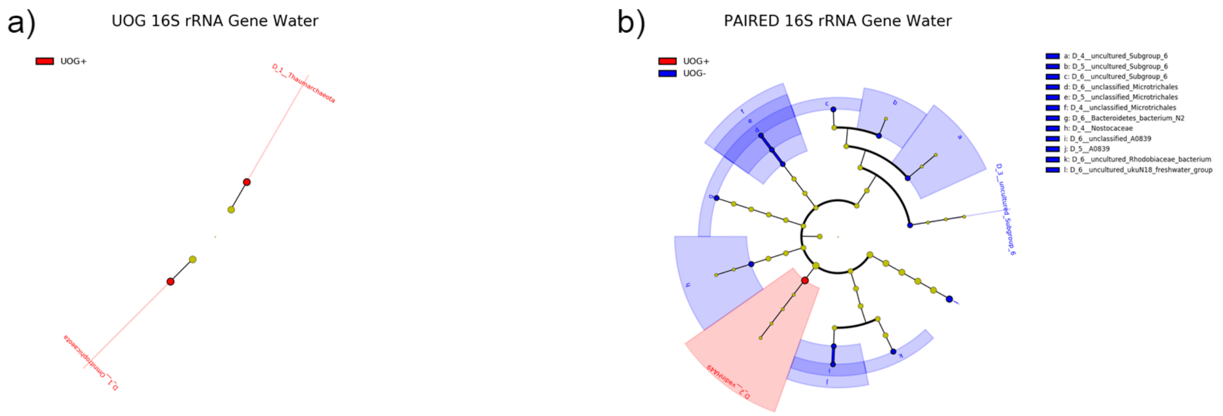

Supplemental Figure 2. Biomarkers of unconventional oil and gas (UOG) activity. A) 16S rRNA gene water, only biomarkers UOG+ were consistently identified by both ALDEx2 and LEfSe, B) 16S rRNA gene PAIRED water dataset, biomarkers of both UOG+ and UOG- were consistently identified by ALDEx2 and LEfSe.

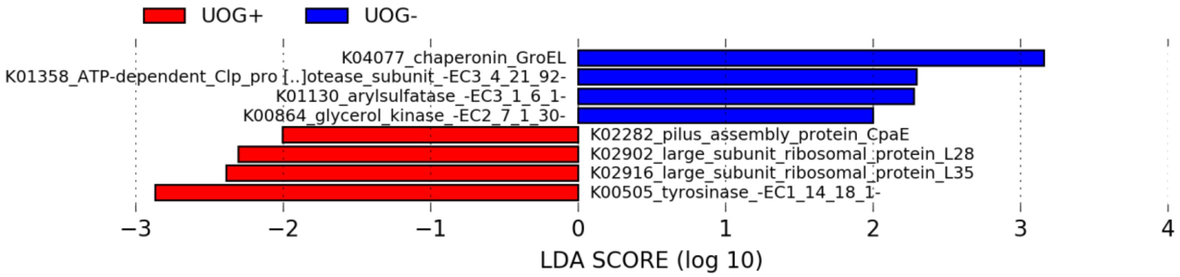

Supplemental Figure 3. Biomarkers of unconventional oil and gas (UOG) activity, general functional genes UOG dataset, biomarkers were identified for both UOG+ and UOG- by LEfSe. The Linear Discriminant Analysis (LDA) score quantifies how well each enriched feature could be used to

differentiate between the two groups. As a result, features that differ more between the two groups have higher LDA scores.

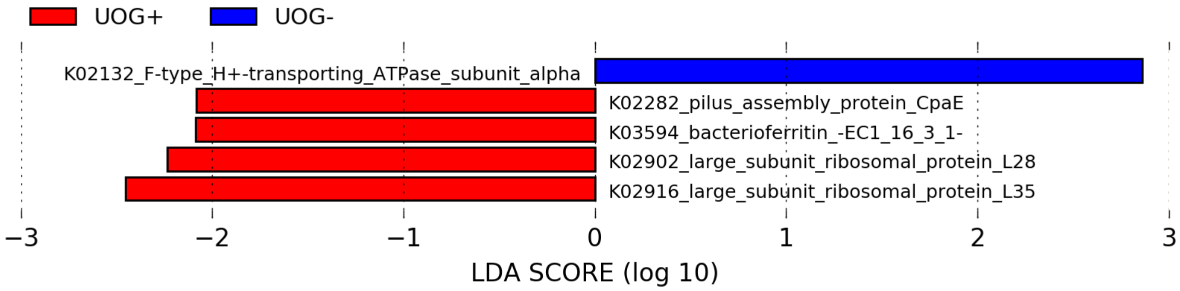

Supplemental Figure 4. Biomarkers of unconventional oil and gas (UOG) activity, general functional genes PAIRED dataset, biomarkers were identified for both UOG+ and UOG- by LEfSe.

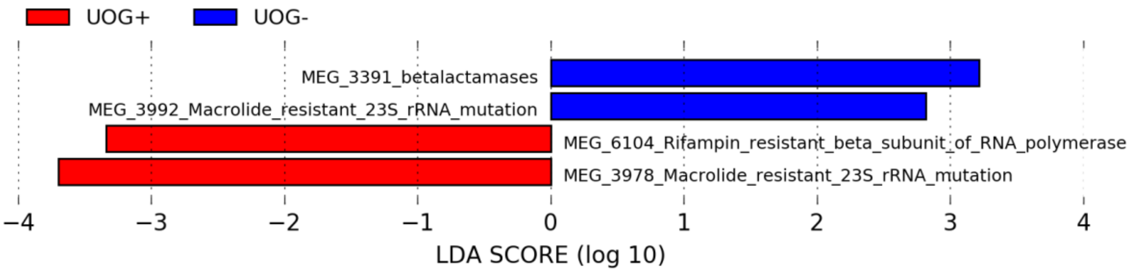

Supplemental Figure 5. Biomarkers of unconventional oil and gas (UOG) activity, ARG UOG dataset, biomarkers were identified for both UOG+ and UOG- by LEfSe.

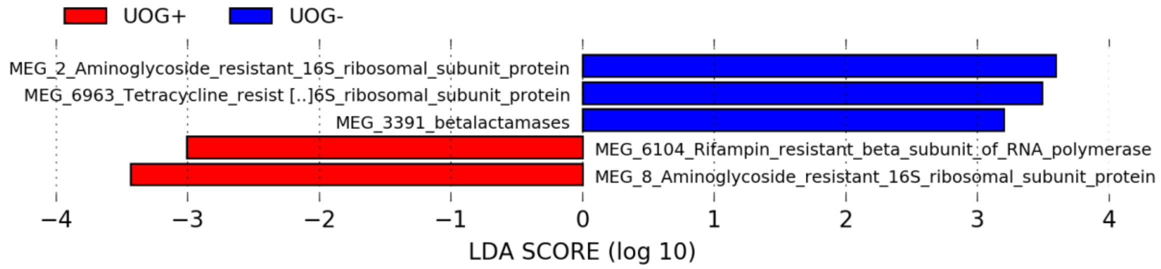

Supplemental Figure 6. Biomarkers of unconventional oil and gas (UOG) activity, ARG PAIRED dataset, biomarkers were identified for both UOG+ and UOG- by LEfSe.

Supplemental References

1. Homer CG, Dewitz J, Yang L, Jin S, Danielson P, Xian GZ, Coulston J, Herold N, Wickham J, Megown K. 2015. Completion of the 2011 National Land Cover Database for the conterminous United States – Representing a decade of land cover change information. Photogrammetric Engineering and Remote Sensing.
2. Moon J-W, Paradis CJ, Joyner DC, von Netzer F, Majumder EL, Dixon ER, Podar M, Ge X, Walian PJ, Smith HJ, Wu X, Zane GM, Walker KF, Thorgersen MP, Poole II FL, Lui LM, Adams BG, De León KB, Brewer SS, Williams DE, Lowe KA, Rodriguez M, Mehlhorn TL, Pfiffner SM, Chakraborty R, Arkin AP, Wall JD, Fields MW, Adams MWW, Stahl DA, Elias DA, Hazen TC. 2020. Characterization of subsurface media from locations up- and down-gradient of a uranium-contaminated aquifer. Chemosphere 255:126951.
3. R Core Team. 2021. R: A language and environment for statistical computing. R Foundation for Statistical Computing, Vienna, Austria.
4. Harrell FE. 2017. Hmisc: Harrell Miscellaneous. R.
5. Kassambara A. 2019. Visualization of a Correlation Matrix using "ggplot2." R, Comprehensive R Archive Network (CRAN).
6. Parada AE, Needham DM, Fuhrman JA. 2016. Every base matters: assessing small subunit rRNA primers for marine microbiomes with mock communities, time series and global field samples. Environmental Microbiology 18:1403–1414.
7. Callahan BJ, McMurdie PJ, Rosen MJ, Han AW, Johnson AJA, Holmes SP. 2016. DADA2: High-resolution sample inference from Illumina amplicon data. 7. Nature Methods 13:581–583.
8. Quast C, Pruesse E, Yilmaz P, Gerken J, Schweer T, Yarza P, Peplies J, Glöckner FO.

2013. The SILVA ribosomal RNA gene database project: improved data processing and web-based tools. Nucleic Acids Res 41:D590–D596.

9. Bolyen E, Rideout JR, Dillon MR, Bokulich NA, Abnet CC, Al-Ghalith GA, Alexander H, Alm EJ, Arumugam M, Asnicar F, Bai Y, Bisanz JE, Bittinger K, Brejnrod A, Brislawn CJ, Brown CT, Callahan BJ, Caraballo-Rodríguez AM, Chase J, Cope EK, Da Silva R, Diener C, Dorrestein PC, Douglas GM, Durall DM, Duvallet C, Edwardson CF, Ernst M, Estaki M, Fouquier J, Gauglitz JM, Gibbons SM, Gibson DL, Gonzalez A, Gorlick K, Guo J, Hillmann B, Holmes S, Holste H, Huttenhower C, Huttley GA, Janssen S, Jarmusch AK, Jiang L, Kaehler BD, Kang KB, Keefe CR, Keim P, Kelley ST, Knights D, Koester I, Kosciolek T, Kreps J, Langille MGI, Lee J, Ley R, Liu Y-X, Loftfield E, Lozupone C, Maher M, Marotz C, Martin BD, McDonald D, McIver LJ, Melnik AV, Metcalf JL, Morgan SC, Morton JT, Naimey AT, Navas-Molina JA, Nothias LF, Orchanian SB, Pearson T, Peoples SL, Petras D, Preuss ML, Pruesse E, Rasmussen LB, Rivers A, Robeson MS, Rosenthal P, Segata N, Shaffer M, Shiffer A, Sinha R, Song SJ, Spear JR, Swafford AD, Thompson LR, Torres PJ, Trinh P, Tripathi A, Turnbaugh PJ, Ul-Hasan S, van der Hooft JJJ, Vargas F, Vázquez-Baeza Y, Vogtmann E, von Hippel M, Walters W, Wan Y, Wang M, Warren J, Weber KC, Williamson CHD, Willis AD, Xu ZZ, Zaneveld JR, Zhang Y, Zhu Q, Knight R, Caporaso JG. 2019. Reproducible, interactive, scalable and extensible microbiome data science using QIIME 2. Nature Biotechnology 37:852–857.

10. Katoh K, Standley DM. 2013. MAFFT multiple sequence alignment software version 7: improvements in performance and usability. Mol Biol Evol 30:772–780.

11. Price MN, Dehal PS, Arkin AP. 2010. FastTree 2 – Approximately Maximum-Likelihood Trees for Large Alignments. PLOS ONE 5:e9490.

12. Paulson JN, Stine OC, Bravo HC, Pop M. 2013. Differential abundance analysis for microbial marker-gene surveys. 12. Nature Methods 10:1200–1202.
13. Faith DP, Baker AM. 2007. Phylogenetic diversity (PD) and biodiversity conservation: some bioinformatics challenges. Evol Bioinform Online 2:121–128.
14. Pielou EC. 1966. The measurement of diversity in different types of biological collections. Journal of Theoretical Biology 13:131–144.
15. Wickham H. 2016. ggplot2: Elegant Graphics for Data Analysis. R, Springer-Verlag, New York.
16. Kassambara A. 2020. rstatix: Pipe-friendly Framework for Basic Statistical Tests in R. R.
17. Lozupone CA, Hamady M, Kelley ST, Knight R. 2007. Quantitative and Qualitative β Diversity Measures Lead to Different Insights into Factors That Structure Microbial Communities. Appl Environ Microbiol 73:1576–1585.
18. McMurdie PJ, Holmes S. 2013. phyloseq: An R Package for Reproducible Interactive Analysis and Graphics of Microbiome Census Data. PLOS ONE 8:e61217.
19. Quensen J. 2020. ggordiplots. R.
20. Segata N, Izard J, Waldron L, Gevers D, Miropolsky L, Garrett WS, Huttenhower C. 2011. Metagenomic biomarker discovery and explanation. Genome Biology 12:R60.
21. Fernandes AD, Reid JN, Macklaim JM, McMurrough TA, Edgell DR, Gloor GB. 2014. Unifying the analysis of high-throughput sequencing datasets: characterizing RNA-seq, 16S rRNA gene sequencing and selective growth experiments by compositional data analysis. Microbiome 2:15.
22. Andrews S. 2014. FastQC A Quality Control tool for High Throughput Sequence Data. Babraham Bioinformatics.

23. Chen S, Zhou Y, Chen Y, Gu J. 2018. fastp: an ultra-fast all-in-one FASTQ preprocessor. Bioinformatics 34:i884–i890.

24. Wood DE, Lu J, Langmead B. 2019. Improved metagenomic analysis with Kraken 2. Genome Biology 20:257.

25. Zhang J, Kobert K, Flouri T, Stamatakis A. 2014. PEAR: a fast and accurate Illumina Paired-End reAd mergeR. Bioinformatics 30:614–620.

26. Rognes T, Flouri T, Nichols B, Quince C, Mahé F. 2016. VSEARCH: a versatile open source tool for metagenomics. PeerJ 4.

27. Huerta-Cepas J, Forslund K, Coelho LP, Szklarczyk D, Jensen LJ, von Mering C, Bork P. 2017. Fast Genome-Wide Functional Annotation through Orthology Assignment by eggNOG-Mapper. Molecular Biology and Evolution 34:2115–2122.

28. Huerta-Cepas J, Szklarczyk D, Heller D, Hernández-Plaza A, Forslund SK, Cook H, Mende DR, Letunic I, Rattei T, Jensen LJ, von Mering C, Bork P. 2019. eggNOG 5.0: a hierarchical, functionally and phylogenetically annotated orthology resource based on 5090 organisms and 2502 viruses. Nucleic Acids Research 47:D309–D314.

29. Camacho C, Coulouris G, Avagyan V, Ma N, Papadopoulos J, Bealer K, Madden TL. 2009. BLAST+: architecture and applications. BMC Bioinformatics 10:421.

30. Doster E, Lakin SM, Dean CJ, Wolfe C, Young JG, Boucher C, Belk KE, Noyes NR, Morley PS. 2020. MEGARes 2.0: a database for classification of antimicrobial drug, biocide and metal resistance determinants in metagenomic sequence data. Nucleic Acids Research 48:D561–D569.

31. Sørensen TJ. 1948. A method of establishing groups of equal amplitude in plant sociology based on similarity of species content and its application to analyses of the vegetation on

Danish commons. I kommission hos E. Munksgaard, København.

32. Shen W, Ren H. 2021. TaxonKit: A practical and efficient NCBI taxonomy toolkit. Journal of Genetics and Genomics https://doi.org/10.1016/j.jgg.2021.03.006.
